# Identifying infection prevention and control gaps in healthcare facilities operating in Rivers state during the EVD outbreak in Nigeria 2014

**DOI:** 10.1186/2047-2994-4-S1-O11

**Published:** 2015-06-16

**Authors:** TJ Okwor, C Tobin-West, O Oduyebo, N Anayochukwu-Ugwu, O Adebola, F Shuaib, O Idigbe, F Ogunsola

**Affiliations:** 1 Community Health, Lagos University Teaching Hospital, Lagos, Nigeria; 2 Community Health, University of Port Harcourt Teaching Hospital, Port Harcourt, Nigeria; 3 Microbiology, Lagos University Teaching Hospital, Lagos, Nigeria; 4 NFELTP, NFELTP, Abuja, Nigeria; 5 NIMR, Nigerian Institute of Medical Research, Lagos, Nigeria

## Introduction

The rate of infection of health care workers in the recent Ebola Virus Disease (EVD) outbreak in West Africa, including Nigeria has shown that the disease can be transmitted and amplified in healthcare settings especially when infection prevention and control (IPC) procedures are ineffective or ignored.

## Objectives

The aim of this study was to evaluate the level of infection control measures operating in health facilities in Rivers state as at the time of the Nigerian 2014 EVD outbreak.

## Methods

The descriptive cross sectional study was done in Rivers state one of the two states that experienced the EVD outbreak in Nigeria. Information on IPC materials and practices in health facilities in the state was collected using the Infection Control Assessment Tool (ICAT) between September 4^th^ and 11^th^ 2014. Each response was scored between 0-3. An overall score of ≥75% was graded as good while <75% was poor. Data was analysed with SPSS Version 20. Ethical statement; this study was conducted during the outbreak of EVD in Nigeria as part of response activities by the members of the Ebola Emergency Operation Center.

## Results

Two tertiary, twenty four public secondary and sixty six private secondary health facilities were studied. Only one of the tertiary healthcare facilities had an IPC committee in place with a focal officer in charge. Only one hospital had an IPC policy which was not operational. None of the health facilities had a good score for both IPC materials availability and practice.

## Conclusion

Gaps existed in IPC in health facilities in Rivers State, Nigeria at the outbreak of EVD. It is recommended that appropriate policy for maintaining and sustaining infrastructure and supplies essential to IPC in health facilities be developed and implemented in all health facilities. Regular surveillance should be put in place to ensure that these items are always available, while hospital administrators and departmental heads must provide the necessary resources to facilitate compliance.

## Disclosure of interest

None declared.

